# Characterization and Evaluation of Controlled Antimicrobial Release from Petrochemical (PU) and Biodegradable (PHB) Packaging

**DOI:** 10.3390/polym10080817

**Published:** 2018-07-25

**Authors:** Alexey Iordanskii, Anna Zhulkina, Anatoliy Olkhov, Sergey Fomin, Andrey Burkov, Mikhail Stilman

**Affiliations:** 1 Semenov Institute of Chemical Physics, Kosygin Str. 4, 119991 Moscow, Russia; annazhulkina@gmail.com (A.Z.); aolkhov72@yandex.ru (A.O.); 2 Polymer Chemistry Department, Plekhanov Russian University of Economics, Stremyannyy Pereulok, 36, 115093 Moskva, Russia; 3 Vyatskiy State University, Moskovskaya ul. 36, 610000 Kirov, Russia; rubberzerg@gmail.com (S.F.); andrey_burkov@mail.ru (A.B.); 4 Mendeleev University of Chemical Technology of Russia, 9, Miusskaya sq., 125047 Moscow, Russia; shtilmanm@yandex.ru

**Keywords:** active packaging, antimicrobial agent, diffusion, controlled release, petrochemical polyurethane, microbial poly(3-hydroxybutyrate), biodegradable packaging

## Abstract

The academic exploration and technology design of active packaging are coherently supplying innovative approaches for enhancing the quality and safety of food, as well as prolonging their shelf-life. With the object of comparison between two barrier materials, such as stable petrochemical polyurethane (PU), (BASF), and biodegradable natural poly(3-hydroxybutyrate) (PHB), (Biomer Co., Krailling, Germany), the study of antibacterial agent release has been performed. For the characterization of polymer surface morphology and crystallinity, the scanning electron microscopy (SEM), atomic force microscopy (AFM) and differential scanning calorimetry (DSC) were used respectively. The antimicrobial activity of chlorhexidine digluconate (CHD) has been estimated by the Bauer–Kirby Disk Diffusion Test. It was shown that the kinetic release profiles of CHD, as the active agent, in both polymers, significantly differed due to the superposition of diffusion and surface degradation in poly(3-hydroxybutyrate) (PHB). To emphasize the special transport phenomena in polymer packaging, the diffusivity modeling was performed and the CHD diffusion coefficients for the plane films of PU and PHB were further compared. The benefit of active biodegradable packaging on the base of PHB is discussed.

## 1. Introduction

Currently, world-wide plastic packaging production exceeds 80 millions tons annually [1]. Most of them originate from fossil resources and are disposed of in landfill after a relatively short period of services. For conventional synthetic polymers, the resulting waste can quickly pile up, leading to a serious environmental harm. Besides, petrochemical packaging fabrication and following incineration cause air pollution due to CO_2_ release in addition to other health public risks [2]. The environmental concern of packaging pollution can be strongly diminished by using renewable natural feedstock polymers and transferring to biodegradable packaging. Therefore, a most advantageous strategy in food pack industry is the design of sustainable barrier polymers [3] which are fully biodegradable in soil and aqueous media, without any negative impact upon environment and living systems.

A distinct boundary separating all known polymers into natural and synthetic classes is quite difficult to lay down. For instance, the non-biodegradable polyethylene that was synthesized from corn-produced ethanol and via natural fermentation is not strictly synthetic material [4]. On the other hand, the production of lactic acid is more often performed through enzyme technologies; however, as was shown in the recent works [5,6], biodegradable polylactide (PLA) could be obtained via synthesis using petrol-based chemicals. As highlighted by Gomez & Michel [6] on the basis of origination and microbial digestion method, the packaging plastics could be categorized into four subdivisions, such as petro-originated conventional, really biobased, enzymatically biodegradable, and biodegradable-biobased plastics. In the cohorts of packaging materials, the segmented polyurethanes (PU) are typical representatives of the first subdivision, and poly(3-hydroxybutyrate) (PHB) belongs to the fourth subdivision in the framework of the above classification.

The family of polyurethanes (PUs) represents the petro-chemically originated synthetic polymers that carry urethane groups (–NH–COO–) in the polymer backbones and that are formed by isocyanate and diol blocks in different combinations. Owing to the structural diversity of the building blocks, their exploitation characteristics are effectively tailored and can be used in the various fields of industry, from constructional materials to biomedical devices. The last decades were marked by their widespread medical applications as catheters, blood lines, constituent elements of artificial heart, orthopedic and dental implants, and many others. Currently, bio-inert and non-biodegradable PUs are efficiently used in drug release matrices and polymeric prostheses for long-term exploitations. The controlled release of chlorhexidine diacetate from medical grade PU films was first communicated in [7].

By analogy with wide medical applications, the potential usage of thermoplastic PUs as active packaging materials is of significant interest because of their processing versatility, reasonable mechanical characteristics, and high barrier properties. For example, the atmospheric gases except for CO_2_, have low permeability through the PU films of Series 11 (BASF) [8], as illustrated by the data in Table 1:

To use the PUs as active packaging materials with a control release function, additional investigations were made for their structural characteristics and especially kinetic data on active agent delivery, which is coherently associated with diffusion in the polymer matrix.

Together with polysaccharides, polypeptides, polynucleotides, and polyisoprenoids, in nature there is one more class of biodegradable bio-based plastics, namely poly(hydroxyalkanoate)s (PHAs) [9,10]. PHAs are dominantly linear polyesters consisting of 3-hydroxy acid monomers linked by the ester bonds in the macromolecules, and accumulated by wide variety of microorganisms as a carbon source and for energy storage. This group of biopolyesters involves about 150 representatives of homo- and copolymers with chemically different pendant groups [11], One of the important advantages of PHAs is determined by the facts that for a benchmark and pilot biotechnology, there are many different renewable substrates including industrial food by-products, oils, lignocellulose, agricultural waste materials, sugars, and biowaste [10,12], so PHAs production does not practically depend on oil and gas production, or on hydrocarbon prices.

The further analysis of interactions between the basic representative of PHAs, PHB, and living or environmental media has shown that along with the listed above principal advancements, there are a number of additional positive characteristics which play an important role under tailoring biodegradable barrier materials for food packaging production. Here we should point out the following encouraging issues:

- the possibility for using food industrial and agricultural wastes as substrates for a biotechnological PHB production that improve environment situation and approaches this biotechnology maximally to the “green” industry category;
- the intermediate products of PHB biodegradation, e.g., 3-hydrobutiric acid, are involved in the biological Krebs cycle, and therefore do not reveal toxicity or an immune response in living systems;
- the terminal groups of its biodegradation in soil or aqueous media are water and carbon dioxide;
- microbial technology does not involve harmful chemicals such as initiators, catalyzers, toxic monomers, and other chemical reagents which accompany the synthesis of petropolymers; therefore the modern biotechnology of PHB and PLA packaging manufacture satisfies EU and USA legislation, taking into account toxic agent migration from polymer packaging to interior food areas [13];
- molecular stereoregularity and hence high crystallinity with perfect crystalline entities (lamellas, spherulites), but here it is appropriate to note that the high crystallinity degree negatively affects the mechanical behavior of PHB;
- processability due to thermoplasticity and good solubility in organic solvents that promotes versatility to produce films, nanofibers and nanoparticles; besides, the conditions for blending with petrochemical and natural polymers;
- the potential for controlled biodegradation and addressing controlled release.

Biocompatibility observed at therapeutic and clinical levels allows PHB to be applied as prosthesis, heart valves and stents, bone drug release systems, surgical threads, fibrillar implants, scaffolds in tissue engineering, and other uses [14,15,16,17,18]. Importantly, all the representatives of PHAs, including PHB, are biocompatible with cells and tissues, and hence PHB-based food packaging can be in long-term contact with meat, fish and other food components without tangible damage. According to [19], PHB was used as an eco-friendly binder in paints. The other applications of PHB-based materials and articles include Tetra Park covers, disposable bags, caps, plates, and cosmetic flacons [10,15,20].

A series of targeting publications provided recently have devoted to the exploration of barrier and mechanical characteristics of the biopolymer [10,21,22,23] All of them have shown that PHB has ample opportunities in passive packaging applications. Additionally, atmospheric gas permeability through the PHB barrier [24], meat salad preservation during pasteurization [25], safe storage of such fat-containing foods such as margarine, mayonnaise, and cream cheese [21], and other food packaging outputs have attested to the high potential of PHB in the food packaging field, either in its pristine form or as the multilayered laminates [24].

Recently, a number of chemical processes, such as epoxidation, chlorination, carboxylation, and others, aimed at modifying PHA molecules, have been used [26,27] with the purpose of giving them additional functional properties. Along with the traditional chemistry of polyesters, here it should be especially noted that the advanced methodologies, namely pegylation [27,28] and click chemistry [28,29], developed over the past few years to promote the design of intelligence packaging materials with variable hyrophilicity and thermoresponsive characteristics. 

The preparation of the academic basis and technological design for active and stimuli-responsible (intelligent) packaging is involved in coherently supplying innovative approaches for sharply enhancing the quality and safety of foods, as well as prolonging their shelf-life [30,31,32,33]. The emerging concept of controlled release for antibacterial agents in biodegradable polymer packaging is currently devoted to improving the passive characteristics of contemporary barrier materials and pack articles.

Direct formulation such as appending active chemicals right into food is a custom technological procedure accompanied by certain shortages. The principal shortage of this process includes overdosing that could particularly arise from food heterogeneity and cause a risk of uneven antibacterial agent distribution. Chemical risk assessment is central to the decision-making process for approving the use of additives that is controlled and legislated by the US FDA and the EU Threshold of Toxicological Concern [13]. Besides, an initially-loaded content of remedies in a food medium to disrupt microbial flora often results in a gradual decrease in antimicrobial content that may lead to nascence of the antimicrobial-resistant mutants [34]. To overcome the above disadvantages, innovative polymer packaging on the base of PHAs and specifically PHB has emerged on the basis of remedy-controlled release that has been successfully invented for biomedical devices [35,36]. In packaging, a preliminary loaded active agent should be delivered into food, thereby enhancing the food quality and the shelf-life simultaneously.

## 2. Materials and Methods

### 2.1. Materials

Chlorhexidine digluconate (CHD) (CAS no 18472-51-0) Medicine Grade was obtained from Xi’an Tian Guangyuan Biotech Co. (Shenyang, China) in a powder form. The powder was diluted with tetrahydrofuran (THF) (CAS no 109-99-9, Merck KGaA, Darmstadt, Germany) to a series of solutions from 0.5 to 10 wt %. The granules of commercially available Elastollan 1154 D (BASF, Florham Park, NJ, USA) (PU) were dissolved in tetrahydrofuran to obtain a 4.0% (*w*/*v*) solution. PHB (Biomer, Krailling, Germany) was dissolved in chloroform (3 wt % solution). All the components were used without special purification. The polymer characteristics are represented in Table 2.

To prepare the PU films loaded with 0.25, 0.5, 0.75, 1.0, 1.25, 2.5 and 5.0 wt % CHD, both solutions in THF were mixed in the different proportions and then casted on the glass plates for solvent evaporation. Stirring of the liquid organic phases was provided by magnetic bars driven by constant-speed high torque motors. To obtain the loaded PHB films, the solution of CHD was added dropwise to the rigorously stirred solution of PHB and after that the mixture was cast onto the glassy surface and maintained at room temperature until the solvent was completely removed.

To determine the homogeneity of the CHD distribution in PU and PHB films, three 7.5 mm-diameter discs were cut out from different sections of each of the 100 mm diameter polymer sample designated for following release. After complete dissolution of the cut PU disc in THF or the PHB-cut disc in chloroform, the amount of CHD in the 3.0 mL solutions was determined at 255 nm by spectrophotometry (Beckman DU-65, (Parkway, IN, USA)). The solutions prepared with blank PU or PHB were used as standard solutions.

### 2.2. Controlled Release Study

The CHD release from the PU and PHB films was carried out as follows: a rectangular fragment was cut out from the prepared films (~10 mg) were suspended in 50 mL of phosphate buffer medium (pH = 7.4 ± 0.2) at 25 °C under continuous stirring at a moderate speed of 200 rpm in a thermostatically controlled glass flask. To estimate the amount of drug released into aqueous medium, 3 cm^3^ of test aliquot was drawn off with the pipette in specified time intervals and analyzed by UV–VIS spectrometer at the wavelength equals to 255 nm that corresponds to the maximal absorption band of CHD. For every aliquot removed, the same quantity of fresh water/buffer was added. Each measuring was repeated threefold and the averaged value has been used as one experimental point.

### 2.3. Bacterial Inhibition Assessment

Microbiological evaluation of antibacterial activity of CHD loaded in PU was performed in Agar Mueller-Hinton medium (рН 7.4 ± 0.2) in accordance to Approved Standard: M7-A5 as elaborated by the National Committee for Clinical Laboratory Standards. The initial activity of bacterium *Staphylococcus aureus* (ATCC 25923) was 5 × 10^6^ cfu/mL. The PU samples loaded with CHD in the discoid form (with 7.5 mm diameter) were located in the Petri dishes. The anti-microbial effect of CHD loaded in PU discs was tested on agar plates inoculated with *Staphylococcus aureus* using the Bauer–Kirby Disk Diffusion Test. For the certain time, the inhibition radial zones (Z) around the polymer sample were measured and compared with blank PU. The bacterial susceptibility was estimated as the moving radial zone corresponding to bacterial growth inhibition and following death during the CHD release.

### 2.4. Instrumental Methods

DSC was performed with the samples using a TA Instruments model Q20 DSC, New Castle, DE, USA. Samples of the ~10 mg weight were cut and crimped in standard Al pans. The DSC cell was purged with nitrogen during measurements (20 mL/min). The samples were equilibrated at 40 °C then ramped up to 185 °C at the rate 10 °C/min to erase any thermal history and held isothermally for 3 min, cooled to 0 °C at 10 °C/min, and then cycles were repeated. The melting (*T*_m_) and the glass transition temperatures (*T*_g_) were determined from the peak maximum and the inflection point of the second heating scan, respectively. Data were analyzed using TA Universal Analysis v4.5A software, New Castle, DE, USA.

To obtain information on the surface morphology of PHB films, SEM observation was performed on a JSM6510LV JEOL LLC scanning electron microscope (Tokyo, Japan) for samples coated with vapor-deposited gold (Au). The samples were mounted onto an aluminum stud and gold-coated using a sputter (Polaron E5200, Denton Vacuum, Moorestown, NJ, USA) set at 25 mA for 10 s. AFM observation was performed on a Ntegra Prima (NT-MDT, Spectrum Instruments. Zelenograd, Russia) directly for uncoated samples. The AFM observation was carried out in the dynamic mode for the initial and phosphate buffer exposition surfaces which had been fixed on the sample holder by using double adhesive tape. Topographic images of square areas of 18 µm × 18 µm were acquired with microlever CSG01 Spectrum Instruments. Zelenograd Russia fabricated from low-stress silicon nitride with a spring constant of 0.03 N/m and nominal radius of 10 nm.

### 2.5. Statistics

**Figure 1:**
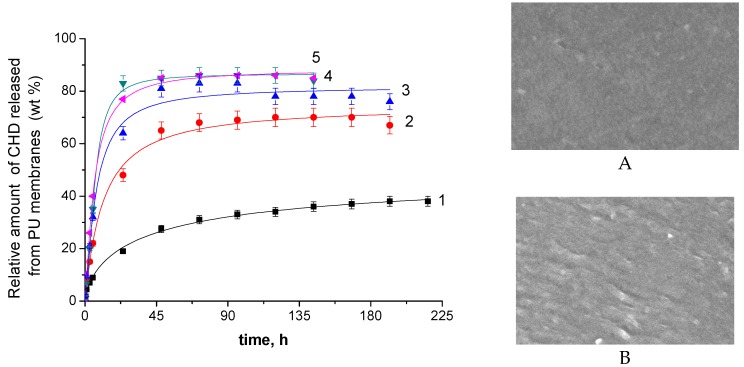
Controlled release profiles of chlorhexidine digluconate (CHD) from polyurethane membranes Elastollan 1154 D (BASF) at room temperature and with different initial loading: 0.25% (line 1), 0.63% (line 2), 1.25% (line 3), 2.5% (line 4) and 5.0% (line 5). A and B are the scanning electron microscopy (SEM) images of the PU surface before and after the CHD release respectively at magnification 1000×.

Precision was been calculated for all of the experimental points on the kinetic curves in Figure 1, Figure 3 and Figure 5. It was represented as the relative standard deviation (RSD.) for three independent measurements. For the kinetic curves of CHD release (Figure 1 and Figure 3), the RSD values vary in the range 3.5–6.4%. The statistical precision depended on the CHD concentration in the polymers. In the initial content interval (≤0.75 wt %) the RSD values did not exceed 4.3%, while at higher CHD concentration (>0.75 wt %) they increased with the antibacterial agent concentration. The samples of the statistical bars (RSD) application were shown in Figure 1. For the lines in Figures 3 and 5 these features were omitted for the sake of clarity. The influence of polymer nature on the statistical deviations was detected. The film thicknesses were equal to 70 ± 2.2 µm with accuracy 0.0314 for both of the polymers.

## 3. Results and Discussion

### 3.1. Controlled Release from PU Films

**Scheme 1:**
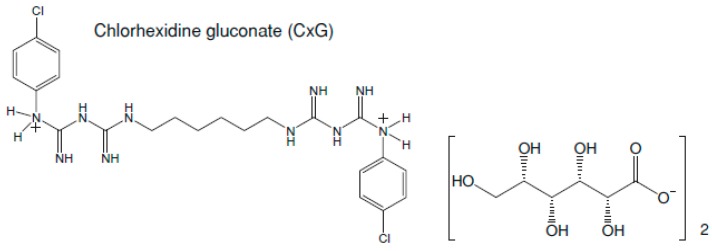
The molecule of CHD with two positive *p*-chlorophenyl guanide groups and two ionized molecules of digluconate as corresponding negative conterions.

In an aim to meet the requirements mentioned in the Introduction, the controlled release system on the base of polyurethane films (Elastollan 1154 D (BASF)) loaded with chlorhexidine digluconate (CHD) was been investigated. CHD has regularly used as an antiseptic with wide antibacterial activity against Gram-negative and Gram-positive bacteria in particular. Its remedial activity derives from two positive charges included in cationic quaternary nitrogen moieties which interact with the phosphate groups of lipopolysaccharides and destroy the corresponding cell membranes, thus killing microorganisms [37]. The formula of the compound given is presented in the Scheme 1.

CHD is readily soluble in aqueous media that enhances its controlled release from a packaging material into food tissue areas such as juicy fruits and fresh meat. Despite the fact that the antiseptic given has been widely used in medicine as a loading chemical in surgical catheters, short-term implants, in dentistry and for oral mucosal delivery [38], its application in the packaging is still fairly limited. Nowadays, for the innovative design of active barrier materials where CHD provides the antibacterial property and simultaneously could progressively migrate into mimic or real foodsas the controlled-delivery agent, there are several polymer systems, namely the porous hybrids of silica [39], paperboard packaging modified by microfibrillar cellulose [40], β-dextrin-cellulose barrier complexes [41], and nano-structured fibrillar cellulose as a coating [42,43], or as nanocrystalline matrices [44].

For the study of controlled release profiles of CHD, PU films with the different antiseptic content in the interval 0.25–5.0 wt % were used. Based on the results obtained, the release kinetics has been recorded as experimental points reflecting antiseptic release from PU films of constant thickness, ~70 µm. Below, in Figure 1, the corresponding release profiles are depicted.

**Figure 2:**
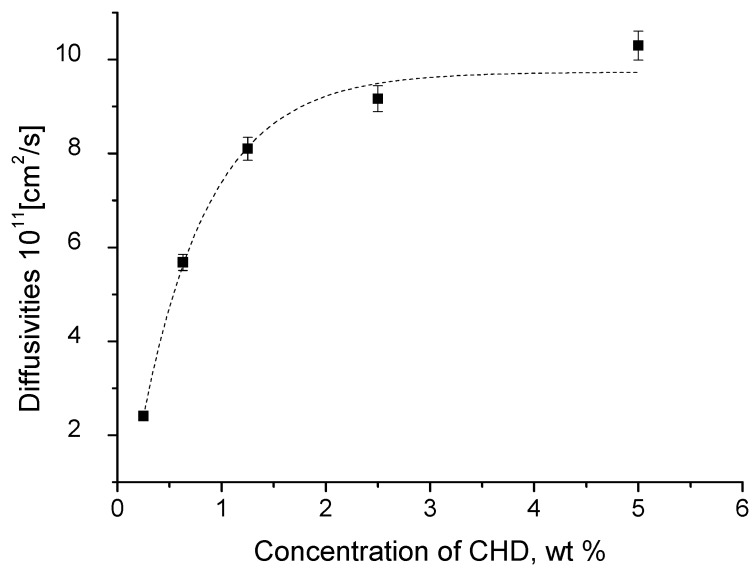
CHD diffusivities in PU Elastollan 1154 D membranes (the rectangular points). The dashed line is the polynomial approximated curve for the averaged effective diffusivities.

Among (bio)chemically stable plastics, for the elastic polyurethane Elastollan R1154D (BASF) [45] that retain their initial sizes, e.g., film thickness, the CHD release kinetic curves have monotonic classical profiles in the coordinates of diffusion (*M*_t_/*M*_∞_ ~ *t*^0.5^) [46]. Here *M*_t_ and *M*_∞_ are the cumulative amounts of active species released at time t, and infinite time, respectively. Under the restricting condition *M*_t_/*M*_∞_ < 0.5, the initial range in each of desorption curves allows us to calculate the effective diffusion coefficients of CHD. The diffusivities presented in Figure 2 are used for the estimation of antiseptic release efficacy and the evaluation of migrant concentration directly in the food area at a certain time, namely the presented CHD dose that aims to repress the pathogens.

As a result of the casting, in the nonporous solid matrix of PU, the antibacterial agent is trapped by the macromolecules and is not be capable of migrating out into a food medium. Owing to atmospheric humidity and/or a water counter-diffusion directed from a fresh food area into the polymer packaging, the PU matrix is slightly plasticized by water molecules. As a result, the segmental mobility is enhanced and the CHD diffusivity is increased as well. The details of water diffusion into polyurethanes with the variable ratio of isocyanate and ester groups have been published by the authors previously [47]. According to a constitutive theory of free volume [48], diffusivity in an excipient matrix is exponentially decreased with an increase in active agent size. In particular, as shown in the work of Lavoine et al. [43], the diffusivities of caffeine and CHD in nanofibrillar cellulose coating differ significantly in accordance with their molecular weights (194 and 898 g/mol respectively). In a similar way, Cozzolino and coworkers [49] performed a comparison of migration rates for the caffeine and an antibacterial protein, lysozyme. The comparison clearly indicated that diffusion of the former was analogously faster than diffusion of the latter (3 × 10^−9^ cm^2^/s relative to 2 × 10^−11^ cm^2^/s correspondingly), owing to a big distinction in the size between low molecular caffeine and the protein. Also, as it was noted rightfully by the authors [43,50] the ionization and the corresponding charge of mobile remedy molecules affected the rate of diffusion in barrier plastics. During the migration of CHD in the PU matrix containing the urethane groups, they could readily interact with biguanide groups of CHD through the hydrogen bonds and thereby slow dawn antiseptic diffusion.

The profiles of controlled release, namely their limit values, showed clearly that there were two populations of CHD in the PU matrix. One portion was capable of migration and was released into aqueous environment, while the other was tightly immobilized in the polymer without participation in delivery process. The ratio between mobile and immobile concentrations increased with the initial loading of CHD in the interval 0.25–1.25%, and after 2.5% it was practically constant.

In the same Figure 1, the surface morphology of the PU film loaded by 1.25% CHD was presented for the initial specimen (A) and after 100 h of release (B) respectively. The observed absence of structure development and morphological texture was quite typical for the PU composed of non-crystallizing blocks of methyldiizocyanate and oligoester diol. It rather reflected the unstructured state of the glass substrate on which the film was formed during solvent evaporation. As a result of subsequent exposure of the sample into the buffer solution it is possible to see the minor alterations in the PU surface pattern on its surface, likely provided by CHD release. The absence of surface porosity simplified the mathematical record of boundary conditions for diffusion equation and facilitated diffusivity evaluation in the framework of a classical Fick’s model.

**Figure 3:**
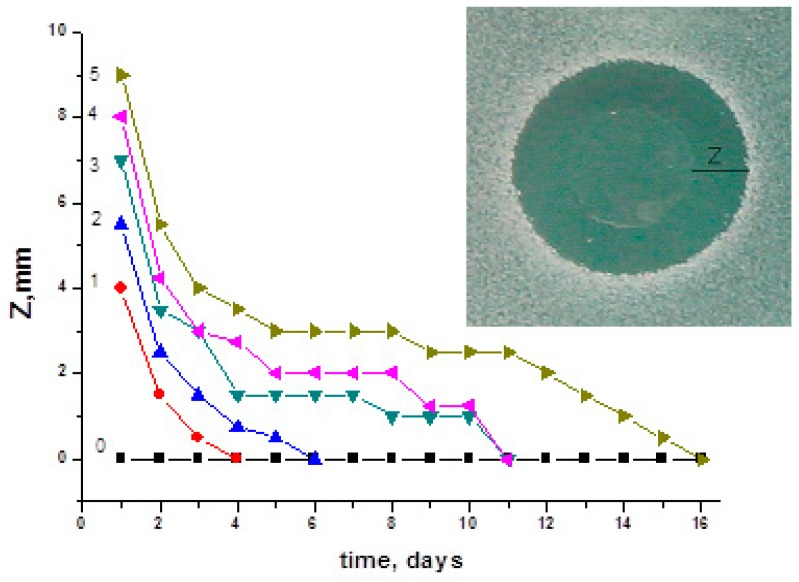
The moving front of antibacterial zone formed by antiseptic CHD at different loading concentrations: 0 (line 0), 0.25 (line 1), 0.63 (line 2), 1.25 (line 3), 2.5 (line 4) and 5,0 (line 5) wt % (gratefully acknowledging to Dr. E. Zhukova for presented microbiological data).

Figure 3 indicates the moving front of antibacterial inhibition zone (Z) for every PU sample located in the center of the agar gel medium, in accordance with the standard Kirby-Bauer method [51]. From this figure it is follows that the strongest antibacterial effect was observed in the first 80–100 h, and then the action of CHD was sharply decreased and progressively finalized in about 11–16 days).

The comparison of controlled release profiles (Figure 1) and kinetics of the moving front (Figure 3) for the antibacterial agent showed that the completion time of antibacterial activity was markedly different, so that the prolonged effect of the polymer was more pronounced in the case of CHD delivery in the agar medium. With a certain degree of probability, this discrepancy can explicate at least by two factors. Firstly, at the border PU-agar the viscous gel medium creates an additional resistance, and external diffusion should be taken into account as the factor retarding the total rate of delivery [52]. Summarizing, the experiments on drug release and gel diffusion test were performed at different boundary conditions of CHD diffusion. Secondly, there is a time gap that separates the moment of initial contact the antibacterial agent with the pathogenic cell and its final destruction [53]. The gap is coherently related to the vital activity of pathogens which resist the moving front spreading and hence influence on the inhibition zone kinetic.

### 3.2. Controlled Release Features for Poly(3-hydroxybutyrate)

For the active biodegradable polymer packaging, the appropriate description of antibacterial agent delivery is a challenging work because, along with transport phenomena, there are destruction processes which can be implemented by hydrolytic or/and enzymatic mechanisms. In addition, the effects of a number of physical and chemical factors should be taken into account. The most important factors are the sorption of water as the main hydrolytic agent [54,55,56]; the degradation of chains [57,58] and, as a consequence, a decrease in polymer molecular weight [58]; possible surface erosion of the polymer [59]; and the evolution of mechanical and thermal characteristics [60,61]. Complementary difficulty in description of these systems is the multilevel structure of the polymer matrix (molecular, nano-structural, crystalline, and microstructural levels) [62]. In this study, the authors explored the behaviour of CHD as the modeling antibacterial agent, to compare two inherent cases of controlled release, namely migration from biodegradable (PHB) and nonbiodegradable (PU) material packaging.

**Figure 4:**
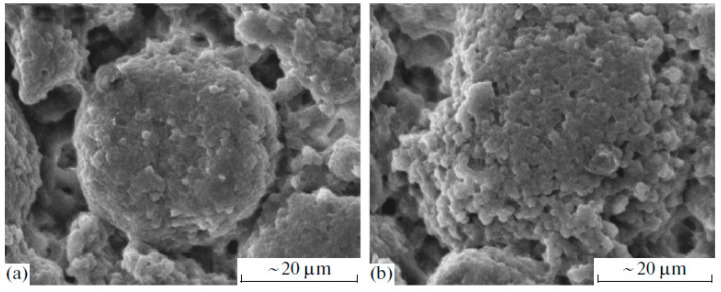
Micrographs of PHB film surfaces. The film was prepared by casting on the glass substrate after gradual evaporation of chloroform [24]. The morphology imiges were obtained for nascent (**a**) and coalescing (**b**) globules.

The morphology of PHB films as revealed by the SEM technique, was composed of two types of globules: spherical globules (Figure 4a) and deformed coalescing globules (Figure 4b). In turn, all polymer microparticles were characterized by the fine surface structure, where smaller spherical elements were sufficiently detectable. The diameters of these elements corresponded to the submicron range and were close to the diameters of natural PHB globules formed in bacteria-produced units of the biopolymer [63].

**Figure 5:**
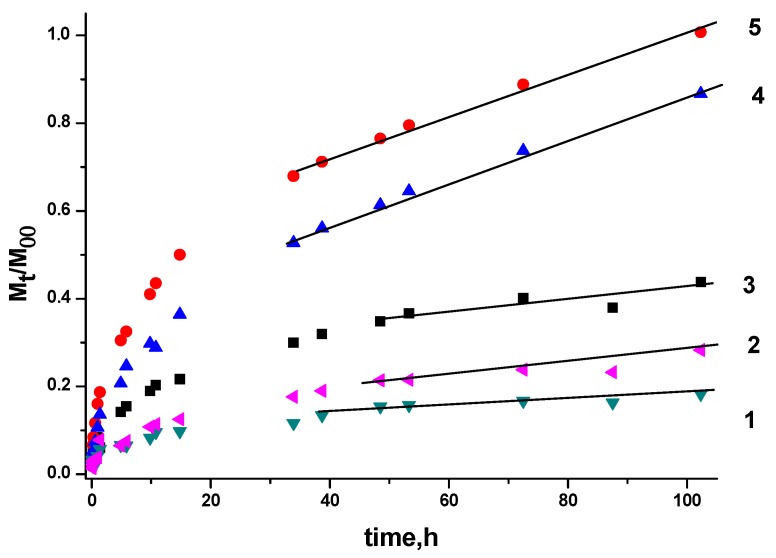
Release profiles for PHB films loaded with CHD at different concentrations: 0.5 (line 1), 0.75 (line 2), 1.0 (line 3) 2.5 (line 4), 5 (line 5) wt %.

Figure 5 shows the typical experimental curve of CHD release from PHB films in the concentration range 5–15 wt %. It was plainly seen that all of the kinetic curves did not a typical character and did not look like the release profiles for PU in the previous section. During the experimental period, all of the curves were monotonically increased without the constant limiting concentration of the antiseptic agent, as it usually occurred in the case of classical diffusion in polymers. Each curve in the figure had a nonlinear initial range and a following linear one. Assuming that the combination of diffusion and hydrolysis is responsible for the release, let us represent a change in its cumulative concentration in the surrounding phosphate buffer, *C_S_*(*t*), at a given moment of time t as a result of two processes: classical diffusion of the remedy from the PHB film, and the surface degradation being typical for hydrophobic polymers [64].

The mathematical description of the antibacterial component desorption from a finite-sized plate (film) with thickness *L* and constant diffusion coefficient *D_S_* was considered in detail in the monograph by Crank [46], where the diffusion equation formulates the Fick’s second law:

(1) $$\frac{{dc}_{S}}{dt}=D_{S}\frac{{d^{2}c}_{S}}{dx}$$

with the initial condition: at *t* = 0 at 0 < *x* < *L* → *c_S_* (0, *x*) = *c_S_*^0^; and the boundary conditions: at *t* > 0 at *x* = 0 and *x* = *L* → *c_S_* (*t*, 0) = *cs* (*t*, *L*) = 0, where *c_S_* (*t*, *x*) is the concentration of the CHD in the PHB and *x* is a coordinate of diffusion.

By averaging the remedy concentrations over the diffusion coordinate x, the intricate solution of Equation (1) is transformed into the simpler expression describing the kinetics of CHD desorption into the surrounding aqueous solution:

(2) $$\frac{G_{S}}{G_{\infty }}={\left[{16D}_{S}t/\pi L^{2}\right]}^{0.5}$$

at condition *G_S_*/*G*_∞_ ≤ 0.5, where *G_S_*(*t*) and *G*_∞_ are the cumulative concentrations of the desorbed compound at time *t* and *t* → ∞ respectively.

Measurements of linear segment slopes for the release profiles of CHD release, *k*, show that their values increase exponentially with the initial loaded concentration *C*^0^_S_ in the PHB:

*k* = *k*_0_·exp(*m*_0_*C*^0^_S_) (3)

where *k*_0_ = (2.7 ± 0.6) × 10^−9^c^−1^ and *m*_0_ = (1.1 ± 0.4) × 10^−2^ g/g

As a result of surface degradation, the antibacterial agent releases from the polymer film into the surrounding solution at a steady state rate with the constant *k*, as is shown in Figure 5, for a long time. At the initial stage of release, on the condition that *G_S_*/*G_S_*^∞^ ≤ 0.5, the process is approximately described by the following equation:

*C_S_* = *k_D_t*^0.5^ + *kt* (4)

where *C_S_* is the current concentration of CHD released from the polymer into solution by time *t*, the diffusion constant equals to 4*G*_∞_[*D_S_*/π*L*^2^]^0.5^, and *k* is the zero-order constant of hydrolysis of PHB molecules, as stated above. Equation (4) determines the total concentration of the CHD transferred from the PHB film into the surrounding solution both via the diffusion mechanism and through the mechanism of surface hydrolysis of the polymer. For a long time, under the inequality: 0.5 < *G_S_*/*G_∞_* < 0.95, the antiseptic release kinetics can be described by the combination of the exponential and linear members of equation [46]:

*C_S_* = {1 − [(8/π^2^)exp(−π^2^*D_S_ t*/*L*^2^)]}*G*_∞_ + *kt* (5)

which is used for evaluating CHD diffusivities in PHB as well.

Equation (5) reflects the relation between the kinetic and diffusion constants, and thus provides another way to evidence that the CHD transport in the system is complicated by the degradation process. As was shown previously in [15,17,65], films, microspheres, ultrathin fibrils of PHB, and its blends with PLA or chitosan slowly degrade in aqueous media in accordance with a zero-order equation. Moreover, recent studies of degradation in a hydrolytic medium or in the presence of enzymes showed that the surface of the biopolymer is involved in the loss of weight of PHB (S-type degradation) [57]. Surface degradation is typical for nonpolar polymer systems because the solubility of water in their bulk is small. Therefore, hydrolysis at the polymer/solution boundary layer, where water molecules easier interact with ester groups of PHB, becomes more probable.

**Figure 6:**
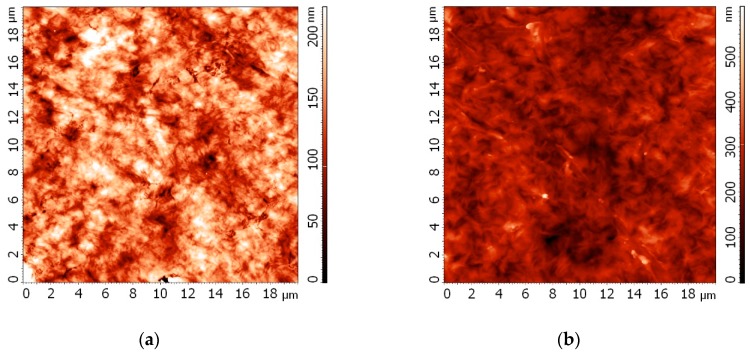
AFM topographic images of PHB films with a scan size of 18 × 18 μm at general magnification ×300: the rough surface of fresh-prepared sample exposed to glass (**a**); the sample exposed to phosphate buffer at 83 days (**b**).

To verify that degradation occurs according to the S-type exactly, the changes in the geometrical profiles of surface depth (roughness) during the contact of the PHB sample with the phosphate buffer solution as a model medium were investigated (see Figure 6). At the initial state, the PHB film has a rough surface with cavities (pores) larger than a micron, as was detected via AFM, and is consistent with SEM data (Figure 4). During exposure in the phosphate buffer, the surface irregularities become smoother and the PHB surface converts into a less-rough surface, owing to partial S-type degradation. Consequently, PHB loses a very small part of its weight via degradation from the surface layer, and along with that, a part of the antibacterial agent that was strongly immobilized in the polymer is released as well, due to surface degradation.

In conclusion for this section, it is worth noting that the performance of two parallel release mechanisms (diffusion and hydrolysis) makes it possible to suggest that as in the case of the polymer system on the base PU, CHD exists in PHB in two populations: the free form with the concentration *G_S_*, which is capable of remedy migration in accordance with Equation (1), and the tightly-bound motionless form. However, in contrast to the nonbiodegradable polymer, the delivery from PHB is provided by the surface degradation of PHB with a kinetic constant *k*. Additional kinetic data on the hydrolytic degradation of PHB films and its derivatives in the absence of antibacterial agent, which is accompanied by a loss in the total weight and rupture of molecular chains and, as a consequence, a decrease in the average molecular weight and a change in the degree of crystallinity were reported in [13] for a deeper degree of hydrolysis for a month and longer.

Comparison of CHD release profiles for two barrier materials such as the stable petrochemical PU and biodegradable natural PHB has shown remarkable differences in the mechanism of antibacterial delivery. Both plastics imitating packaging materials with different origination are active systems, because along with barrier behavior, both of them provide antibacterial function via remedy controlled release. However, only PHB affords the constant rate of CHD delivery (zero-order release) (see Figure 5) that occurs due to the effect of PHD surface degradation. As a result of this, the PHB (in contrast to PU) demonstrates the possibility of steady state profile of antibacterial agent delivery into a food area. Finally, there is one more benefit of PHB promotion, namely the eco-friendly biodegradation of PHB waste packaging under natural conditions. In summary, the combination of principally important features such as barrier behavior, appropriate controlled release profile, and biodegradation without toxic products, is a significant advance for the innovative PHB material as an active biodegradable packaging in the food and agriculture industry.

## 4. Conclusions

In the very near future, biodegradable bioplastics will play a greater role in the packaging service and industry. They have a real opportunity to replace conventional petrochemical plastics, to decrease impacts upon landfill volume, and to involve widely renewable resources. Along with that, it is necessary to make a next important step including the transfer from passive to active packaging materials. In active packaging systems, the barrier function of packages is enhanced by incorporating oxygen and water absorbers, ethylene removers, preservatives, ethylene removers, and antimicrobial agents [66]. One of the principal concerns in food industry is the protection against food deterioration that still represents a global problem of public health. So, five years ago, the Center for Disease Control and Prevention announced that the impact of food-borne diseases in the United States results in 76 million sick people annually, 325,000 of which were hospitalized and 5000 passed away [67]. The urgency in toxicity avoidance demands innovative efforts in the development of antimicrobial materials with remedy controlled release in order to improve food quality, extend shelf life, and retain the food components spoilage.

For the development of active packaging materials, three classes of sustained polymers based on renewable sources are primarily used, namely polysaccharides (chitosan, cellulose, its derivatives and the like) [68], poly-α-hydroxyacides (polylactides, polyglycolides, and their copolymers) [65,69,70,71,72,73], and poly-β-hydroxyacides (polyalkanoates) with their basic representative-poly(3-hydroxybutyrate) [PHB] [24,74,75,76] that have attracted special attention in the framework of this paper. The combination of degradation ability and remedy absorption capacity was an inherent stimulus for this presentation.

## Figures and Tables

**Table 1:** Gas and water vapor permeabilities through the films of polyurethane (PU) Elastollan^R^ (BASF).

Grade	CO_2_	N_2_	O_2_	H_2_	CH_4_	Ar	He	H_2_O
1180 A	230	6	21	70	18	14	50	388
1185 A	180	5	16	60	14	9	40	183

Dimensions: [m^2^/s·Pa]·10^−18^ for all gases and [g/m^2^·day] for water at 23 °C, 85% relative humidity (RH) according to DIN 53122-1.

**Table 2:** Characteristics of synthetic (PU) and natural (PHB) elastomers.

Polymer	*M*_η_, kDa	*ρ*, g/cm^3^	*H*, MPa	ε, %	*T*_g_, °C	*T*_M_, °C	Water Uptake, %	Hydrolysis Resistance	Bioresistance
Elastolan 1154 D, (PU)	350	1.17	30	400	−16	140 *	0.3	high	very high
Biomer Krailing, (PHB)	460	1.25	80	~10	7	175	0.9	poor	very poor

*M*_η_ is viscosity average molecular weight, *ρ* is density, H is tensile strength, ε is elongation at break, *T*_g_ and *T*_M_ are glass transition temperature and melting point respectively, water uptake given at 230 °C and RH = 50%; *—for Elastolan 1154 D, the temperature of softening is presented [20]. For both polymers (PU and PHB), films with thickness within 70 ± 2 µm were used.
